# Invasive Fungal Infections in Children with Acute Leukemia: Epidemiology, Risk Factors, and Outcome

**DOI:** 10.3390/microorganisms12010145

**Published:** 2024-01-11

**Authors:** Tamar Ruth Gal Etzioni, Nurit Fainshtain, Adi Nitzan-Luques, Gal Goldstein, Sigal Weinreb, Violeta Temper, Maya Korem, Dina Averbuch

**Affiliations:** 1 Faculty of Medicine, Hebrew University of Jerusalem, Jerusalem 91120, Israel; tamar.gal2@mail.huji.ac.il (T.R.G.E.); nuritf14@gmail.com (N.F.); adi.nitzanluques@sickkids.ca (A.N.-L.); galg@hadassah.org.il (G.G.); sigalv@hadassah.org.il (S.W.); vtemper@hadassah.org.il (V.T.); mayak@hadassah.org.il (M.K.); 2 Pediatric Division, Hadassah Medical Center, Jerusalem 91120, Israel; 3 The Dyna & Fala Weinstock Department of Pediatric Hematology Oncology, Hadassah Medical Center, Jerusalem 91120, Israel; 4 Department of Microbiology and Infectious Diseases, Hadassah Medical Center, Jerusalem 91120, Israel; 5 Pediatric Infectious Diseases, Hadassah Medical Center, Jerusalem 91120, Israel

**Keywords:** invasive fungal infections, acute leukemia, children, risk factors

## Abstract

Invasive fungal infections (IFI) cause morbidity and mortality in children with acute leukemia (AL). We retrospectively collected data on febrile neutropenic episodes (FNE) in AL children (2016–2021) and assessed factors associated with proven/probable IFI. Ninety-three children developed 339 FNE. Seventeen (18.3%) children developed 19 proven/probable IFI (11 yeast; eight molds). The proven/probable yeast IFI rate was 6/52 (11.5%) in children who belong to the high risk for IFI category (HR-IFI-AL: high-risk acute lymphocytic leukemia (ALL), acute myeloid leukemia, relapse); and 5/41 (12.2%) in the non-HR-IFI-AL category (standard/intermediate risk ALL). The proven/probable mold IFI rate was 7/52 (13.5%) in HR-IFI-AL children and 1/41 (2.4%) in the non-HR-IFI-AL category. In the multivariable analysis, underlying genetic syndrome, oral mucositis, and older age were significantly associated with proven/probable IFI, while a longer time since AL diagnosis was protective. Two of 13 (15.4%) HR-IFI-AL children died because of IFI. The elevated risks of proven/probable mold IFI and the associated mortality in HR-IFI-AL children, and high risk of invasive candidiasis in the non-HR-IFI-AL group, emphasize the need for the close monitoring of local epidemiology and the adjustment of practices accordingly.

## 1. Introduction

Invasive fungal infections (IFIs) are a significant cause of morbidity and mortality in patients with acute leukemia (AL). The primary causes for IFIs are yeasts (mainly candidemia and hepatosplenic candidiasis) and molds (mainly aspergillosis in the lungs, sinuses, or brain) [1]. The rate of IFI risk for children with AL shown in recent studies varies between 4.8 and 18.4% [2,3,4,5,6,7,8]. The main risk factors for its development in these patients are profound and prolonged granulocytopenia (<100 cells/mm3 for 7 days or more), chemotherapy-induced mucositis, and prolonged antibiotic and steroid treatment [1,9,10,11]. Reported mortality for AL patients with IFI in these studies ranges from 17.5 to 68%, and is significantly higher than for AL patients without IFI [2,3,4,7,8]. Early diagnosis of IFI and appropriate treatment improve patient prognosis [10,11].

Based on a combination of diagnostic, clinical, laboratory, and imaging criteria, IFIs are classified as possible, probable, or proven [12]. The three approaches to diagnosis and prevention of IFI are [1,13]: (a) primary prophylaxis: antifungal administration for the duration of chemotherapy; (b) empirical treatment: initiating antifungals following 96 h of febrile neutropenia; and (c) diagnostic-driven: initiation of antifungals following positive biomarkers, such as blood galactomannan and compatible IFI in imaging studies. Pediatric patients with IFI commonly present with non-specific symptoms and imaging. Data on the efficacy of diagnostic studies in this patient population are limited.

Current guidelines recommend primary prophylaxis in patients/centers with IFI rates ≥ 10% [1]. The disadvantages of this approach include adverse effects of antifungals, drug interactions, drug resistance, and its high cost. In populations with lower infection rates, it is therefore acceptable to apply a diagnostic-driven approach. Appropriate strategies are chosen according to each center’s IFI rate and epidemiology. This study assessed IFI rates and epidemiology in children with AL in our tertiary-care hospital and analyzed IFI risk factors during febrile neutropenia (FN) episodes, aiming to select appropriate management of IFI in our institution.

## 2. Materials and Methods

### 2.1. Study Design

In this retrospective observational study, we collected data on all FN episodes in children (<18 years old) with AL treated at the Hebrew University-Hadassah Medical Center (HMC) between 2016 and 2021. HMC is a tertiary medical center that provides care for a population of 475,000 children in Jerusalem and its surroundings, as well as patients referred from around the country and abroad. In patients who underwent hematopoietic stem cell transplant (HSCT) during their oncologic treatment, we documented only episodes that occurred prior to HSCT. For each FN episode, we documented demographic information, comorbidities, and underlying disease-related details, such as age at diagnosis, AL type, and stage of treatment. Additionally, we recorded characteristics for each FN episode, including time since the preceding chemotherapy, episode duration, presence of breakthrough fever, oral mucositis or diarrhea, central line use, and administration of anti-fungal medications. Clinical and imaging characteristics, treatment and outcomes were described for all IFI episodes. The study was approved by the Hadassah Medical Center Ethical Review Board (0830-20-HMO).

### 2.2. Routine Management of Children with Acute Leukemia

Management protocol for fungal infections in HMC’s pediatric haemato-oncology department has evolved over the years. Prior to 2017, there was no clearly established protocol. Children with fever and neutropenia of over 96 hours’ duration were treated empirically with fluconazole, underwent CT scanning of the lungs, sinuses, and abdomen, and began anti-mold treatment at the discretion of the treating physician. In 2017/2018, in response to an impression of relatively high rate of invasive candidiasis, all patients at high risk for IFI (HR-IFI-AL)—including acute myeloid leukemia (AML), high-risk acute lymphoblastic leukemia (HR ALL), or relapse AL—were given prophylactic fluconazole (6 mg/kg/day). Further, the approach to screening and management of invasive molds infection became diagnostic-driven, with twice-weekly serum galactomannan monitoring from FN onset, and CT scanning of lungs, sinuses, and abdomen after 96 hours’ FN, with treatment decisions based on findings. Bronchoalveolar lavage (BAL) was performed in children with abnormal lung CT (if no contraindications were present). Samples collected underwent bacterial, mycobacterial, and fungal staining and cultures, galactomannan testing, PCR analysis for respiratory viruses, and PCR and staining procedures for *Pneumocystis jirovecii*. All children received routine prophylaxis with low-dose trimethoprim-sulfamethoxazole from AL diagnosis.

### 2.3. Definitions

- Fever: a single oral measurement of ≥38.3 °C, or a temperature of ≥38.0 °C sustained over a one-hour period [14].
- Severe neutropenia: absolute neutrophil count (ANC) of <500 cells/µL.
- FN episode: first day of episode was the day on which the patient had fever and neutropenia. Last day was the first day on which ANC was >500 cells/microL for at least 7 consecutive days, regardless of fever.
- Breakthrough fever: a new fever during the same FN episode, after at least 48 fever-free hours.
- IFI: presence of invasive fungal infection was defined as possible, probable, or proven based on the EORTC criteria [12].
- High risk/non-HR AL: oncologic risk category was defined according to accepted definitions in the relevant treatment protocols used during the study period [15,16].
- High risk/non-HR for IFI (HR-IFI-AL/non-HR-IFI-AL): risk for IFI was defined according to the eighth European Conference on Infections in Leukaemia guidelines (ECIL-8) [17]. Briefly, AML, relapse AL and high-risk ALL belong to the HR-IFI-AL group, defined as ≥10% risk of proven or probable IFI. Standard- and intermediate-risk ALL belong to the non-HR-IFI-AL.
- Antifungal treatment since beginning of episode (before new IFI diagnosis): antifungal treatment given since the first day of FN episode.

### 2.4. Statistical Analysis

Based on the data collected, the IFI rate in the study population was calculated with a confidence interval (CI) of 95%. Pearson’s Chi-squared test was used to compare our population’s rate with that reported for IFI. IFI rates were shown both as rate per patient and rate per FN episode. For the risk factor analysis, we compared demographics and baseline disease data, including stage of treatment and use of steroids, timing and length of episodes, presence of specific conditions (including breakthrough fever, oral mucositis, and diarrhea), presence of central line, and use of antifungal medications in FN episodes with and without IFI. Data for continuous variables are shown as mean and standard deviation for data that was normally distributed, and median and interquartile range (IQR) for data that was not normally distributed, whereas categorical data are presented in absolute numbers and in percentage of the total population. Associations between two categorical variables were tested with Pearson’s Chi-squared or Fisher’s exact test, and continuous variables between two groups were compared using the two-sample *t*-test or the Mann–Whitney non-parametric test. The Mann–Whitney non-parametric test was used for data that was not normally distributed. The two-sample *t*-test was used for data that was normally distributed. Statistically significant variables found in the univariate analysis were assessed in multivariable analysis using logistic regression with a forward stepwise (likelihood ratio) progression. A two-sided *p*-value of ≤0.05 was considered statistically significant for all statistical tests.

## 3. Results

### 3.1. Characteristics of Children with AL during the Study Period

During the study period, there were 339 FN episodes in 93 children with AL. The background information of these patients is shown in Table S1: 51 of them (54.8%) were girls and 42 (45.2%) boys; mean age at diagnosis was 5.7 years. Sixty-six (71%) had ALL, and 27 (29%) AML. Nine of the children (9.7%) had genetic syndromes.

### 3.2. IFI Episodes

The rates of IFI in all pediatric patients with AL, as well as specifically in those with ALL and AML, are shown in Table S2. IFI episodes are described in detail in Table S3. Twenty-two of the 93 patients (23.7%; 95% CI 15.5–33.6%) developed 25 episodes of IFI; among them, 19 patients experienced one IFI episode, two patients developed two IFI episodes (19, 20 and 21, 22) in two separate FN episodes, and one patient developed two IFI episodes (11, 12) in the same FN episode. The rate of IFI per FN episodes was thus 24/339 (7.1%; 95% CI 4.6–10.4%) (Figure S1a,b).

Seventeen of the 93 children (18.3%; 95% CI 11.0–27.7%) developed 19 episodes of proven/probable IFI during the course of 18 FN episodes, making the rate of proven/probable IFI per FN episode 18/339 (5.3%; 95% CI 3.2–8.3%) episodes (Figure S1c,d).

Twelve children (12.9%; 95% CI 6.9–21.5%) had yeast IFI, 11 of them proven and one possible. The rate of proven yeast IFI was thus 11/93 (11.8%; 95% CI 6–20.2%). *Candida* species was identified in nine of these patients—in six it was non-*albicans*. All tested *Candida* spp. were susceptible to fluconazole. Six of the proven yeast IFIs were in the HR-IFI-AL group (Table 1, Table S3), 3/6 (50%) as a breakthrough infection while on fluconazole prophylaxis, two of them caused by *Candida glabrata*.

Of the 11 patients with proven yeast IFI, eight recovered completely, and three died, all in the HR-IFI-AL category. One of the deaths was in a child (episodes 11 and 12) with probable invasive pulmonary aspergillosis. Treated with voriconazole, 16 days later she developed disseminated candidiasis due to voriconazole-susceptible *C. glabrata*, and died eight days after that. The contribution of these two fungal infections to her death cannot, therefore, be ruled out. The second death resulted from oncologic disease; it was in a child (episode 19) with invasive candidiasis with abdominal dissemination followed by lung mucormycosis (episode 20). The third child (episode 22) had hepatic candidiasis and succumbed to refractory AML.

Thirteen children developed mold IFI (14%; 95% CI 7.7–22.7%), among them four proven, four probable and five possible; the rate of proven/probable mold IFI was 8.6% (95% CI 3.8–16.2%). Among the eight proven/probable cases, two were mucormycosis according to histopathology, one was *Fusarium solani* infection and the other five were invasive aspergillosis. Seven occurred in the HR-IFI-AL group (Table 1, Table S3). Five patients recovered completely from the mold IFI; three, all in the HR-IFI-AL group, died. In one death, the child (episodes 11 and 12) had both mold and yeast infections (described above); the second child (episode 14, referral from abroad) presented with refractory relapsed ALL and disseminated fusariosis, and died after blood cultures became negative on treatment, likely due to relapsed AML; and in the third case, the patient (episode 15) had disseminated mucormycosis and died within days, likely due to the IFI. Thus, two of 13 (15.4%) children with IFI, classified in the HR-IFI-AL group, died as a result of IFI.

Rates of proven or probable IFI according to treatment stage and risk category are shown in Table 1. The rates of yeast IFI were similar in the HR-IFI-AL and non-HR-IFI-AL groups, whereas that of mold IFI was higher in the HR-IFI-AL than in the non-HR-IFI-AL groups (*p* = 0.053).

The distribution of IFI during the study period is presented in Figure S2. A higher number of mold IFIs was observed during 2018/2019—a period when there was construction in the department.

### 3.3. Episodes of FN

Data from the 339 FN episodes and comparison between episodes with and without proven/probable IFI are shown in Table 2.

### 3.4. Comparison of FN Episodes with and without Proven or Probable IFI

The following factors were associated with FN episodes with proven/probable IFI: presence of genetic syndromes, AML, induction chemotherapy, fever at the end of the FN episode, presence of breakthrough fever, presence of oral mucositis or diarrhea, recent use of steroids, and FN episode that developed shorter time since AL diagnosis. There was borderline significance of an association between older age at AL diagnosis with FN episodes with proven/probable IFI (*p* = 0.053).

In the multivariable analysis (Table 3), presence of genetic syndromes, oral mucositis, and older age were significantly associated with proven/probable IFI, while longer time that elapsed since AL diagnosis was a protective factor.

## 4. Discussion

In this study, we describe the epidemiology and risk factors for IFI in a pediatric population with FN episodes associated with AL, within a large tertiary-care center in Jerusalem. Our main findings were a high rate of yeast IFI in non-HR-IFI-AL patients, and high mortality associated with invasive mold infection in pediatric HR-IFI-AL patients.

In 17 (18.3%) children with AL in our study, there were 19 proven/probable IFI episodes, a rate which exceeds those documented in most recent investigations involving children with AL. A multicenter Italian study found the rate of proven/probable IFI to be 4.8% (53 episodes of 1101) in children with AL given antifungal prophylaxis, mainly fluconazole [2]. A multicenter German study, where anti-mold prophylaxis was inconsistently employed, reported IFI incidence as 12.8% (23/179) [5]. An Israeli study reported a rate of 7.8% (50/271) in children with AL, with anti-mold prophylaxis administered in AML only during induction [6]. A recent study in Turkey found the IFI rate in pediatric AL patients as high as 11.1% (34/307) [8]: high-risk ALL patients in this study received anti-yeast prophylaxis, and those with AML or relapse AL were given anti-mold prophylaxis. In addition to the different antifungal prophylaxis policies, differences in IFI rates may be explained by local fungal epidemiology, definition criteria, hospital conditions, and availability of different diagnostic tools. All these studies [2,5,6,8] were based on earlier 2008 EORTC criteria for IFI diagnosis [18], which recommended the use of a 0.5 optical density index as GM threshold—which emphasizes the high rate of IFI that we found, as the galactomannan threshold used in our study, according to 2019 EORTC guidelines [12], was higher.

Specifically, high invasive yeast infection rates were documented in patients traditionally in the non-HR-IFI-AL group (children with standard- and intermediate-risk ALL) (12.2%; 5/41; Table 1) [17]. This conventional classification finds support in a recent international, multicenter clinical trial conducted by AIEOP-BFM, where the reported IFI rate among standard- and intermediate-risk ALL patients was 2.8% (131/4724). In contrast, high-risk ALL patients exhibited a higher IFI rate of 6.6% (93/1403) [19]. Notably, the chemotherapy regimens employed in our cohort of standard- and intermediate-risk ALL patients mirror those outlined in the AIEOP-BFM protocol. It, therefore, seems that the observed differences in IFI rates cannot be attributed to variations in the intensity of chemotherapy regimens. One possible explanation may be use of antifungal prophylaxis in this multicenter study population, administered at the discretion of the treating physician and not reported. Another possible explanation for the high rate we noted could be rigorous diagnostic effort, including imaging and biopsy, that proved IFI diagnosis in three of these five cases. Antifungal prophylaxis is not routinely recommended in this category of patients [17], who are considered low risk for IFI. Our data, however, question whether such prophylaxis is indeed required in this setting. Although none of the non-HR-IFI-AL patients died from IFI, four required prolonged antifungal therapy due to disseminated candidiasis, which could probably have been avoided had prophylaxis been used. Potential prophylaxis b”nefit’ must be weighed against its risks, which include hepatotoxicity, selection of fluconazole-resistant *Candida* spp., and cost.

Half of the invasive yeast infections in patients at high risk for IFI (3/6) occurred while they received fluconazole prophylaxis. One of these three *Candida* spp. was fluconazole susceptible; another was susceptible dose-dependent (S-DD) and the third was *C. glabrata* with no available susceptibility but can be presumed fluconazole S-DD or resistant. It may, therefore, be assumed that our routine fluconazole prophylaxis dose of 6 mg/kg/day may be lower than required for some patients. Recent guidelines recommend a higher fluconazole dose of 8–12 mg/kg/day [17]. The failure of fluconazole prophylaxis reported in earlier randomized controlled trials [20,21] is attributed to profound immune deficiency and severe mucositis in addition to inadequate fluconazole dosage. In accordance with our data, half of IFIs in a multicenter Italian study occurred on prophylaxis, mainly with fluconazole [2].

In HR-IFI-AL patients, we currently use fluconazole prophylaxis combined with a diagnostic-driven approach. This approach was reported to be safe compared with the empirical antifungal therapy administered in a randomized controlled multicenter Chile study in neutropenic children with cancer, with a significant reduction in the use of antifungals; both children at high and at low risk for IFI were included [22]. Antifungal prophylaxis was not used in this study, with the total IFI rate in both groups at 12%. Similarly, a recent study in neutropenic adults receiving fluconazole prophylaxis following treatment for AML, myelodysplastic syndrome (MDS), and allogeneic HSCT demonstrated similar mortality rates and significantly reduced use of caspofungin in patients managed according to a diagnostic-driven vs. an empirical approach. Their rate of IFI was about 7% in both study arms [23]. In a multicenter Australian study, the prevalence of proven/probable IFI during primary AML therapy was 10.3% (95% CI 6.7–15.0%) [24]. In our cohort of HR-IFI-AL patients, the rate of proven/probable IFI was higher (25%; 13/52, Table 1), compared with recent studies involving similar pediatric cohorts. Specifically, 13.5% (7/52) of our HR-IFI-AL patients developed mold IFI (Table 1), and two of these seven patients (28.5%) died of it (Table S3). Six of our mold IFI episodes could potentially have been prevented by posaconazole (Table S3), all except for fusariosis caused by posaconazole-resistant *Fusarium solani*); posaconazole prophylaxis is currently strongly recommended (A-IIt level of recommendation) for HR-IFI-AL patients [17]. Itraconazole or voriconazole (both with B-Iit recommendation) could possibly prevent four or five of these infections, respectively. Neither would prevent two cases with mucormycosis, and itraconazole is ineffective against *Fusarium* spp. Based on current recommendations [17], this high rate of mold IFI in patients in the HR-IFI-AL group, along with high mortality in those who developed mold IFI, justifies anti-mold prophylaxis usage in such patients. Since almost all mold IFI in our center occurred during 2018/2019, when there was construction in the department, with virtually no instances in the study years before and after, a prophylactic approach is warranted during periods of construction in proximity to departments hospitalizing patients at risk [25]. In contrast, the rate of proven/probable mold IFI in patients in the non-HR-IFI-AL group was 1/41 (2.4%). Anti-mold prophylaxis is thus unwarranted in this group of patients.

We found yeast IFI, predominately caused by non-albicans *Candida* spp., was more frequent than mold IFI. IFI epidemiology is known to differ geographically. In both a multicenter international study of children with ALL and a multicenter Australian study in children with AML, the proportion of mold infections was higher than that of yeast [19,24], whereas multicenter Italian and Polish studies found the proportion of yeast IFIs was equal to or higher than molds [2,26]; and that the majority of yeast IFIs (75–90%) were due to non-*albicans* species [2,24,26,27].

The presence of genetic syndromes, short duration from AL diagnosis to FN episode, oral mucositis, and older age at AL diagnosis were all associated in multivariable analysis with increased IFI risk in our study. Data on patients with genetic syndromes are principally based on children with Down’s syndrome. The degree of mucositis, mainly due to methotrexate toxicity, and presence of infection were found to be higher in children with ALL and Down’s syndrome by both the Children’s Cancer Group (CCG) [28] and the Berlin-Frankfurt-Münster group (BFM) [29]. A higher degree of mucositis is, therefore, a possible explanation for an increased IFI rate in children with genetic syndromes. Another factor that can accompany genetic syndromes and contribute to increased IFI rates is possible underlying immune deficiency.

FN episodes occurring shortly after AL diagnosis and the presence of mucositis are known to be associated, as shorter intervals from diagnosis correspond to a more aggressive phase of treatment, usually during induction chemotherapy, leading to prolonged severe neutropenia. Mucositis is generally related to increased IFI risk because it predisposes to fungal translocation from the gastro-intestinal tract and causes a decrease in oral intake, often requiring total parenteral nutrition. Higher IFI rates in the induction phase have been reported [30,31]. One example is a study in Germany [5] of 211 pediatric patients receiving chemotherapy for hematologic malignancy, in whom all 11 documented IFIs were diagnosed during induction or reinduction. In other studies, performed in Germany, Turkey and the UK, the highest risk of IFI was observed during the induction or consolidation phases [8,19,27,32].

Some studies have demonstrated that age above 9–10 years is associated with higher IFI rates [4,5,8,27]. The risk for proven/probable IFI was found to be significantly increased in children with ALL ≥ 12 years of age [OR 1.4 (95% CI 1.3; 1.6)] (*p* < 0.0001) in an international study [19]. In research conducted in California in 1052 children and young adults (under 21 years of age) with hemato-oncological diseases, who did not receive antifungal prophylaxis, patients with IFI were significantly older than those without [7]. This can probably be explained by more aggressive treatments being used in older children and adolescents: 10 years of age is considered sufficient to assign a patient to high-risk AL protocol, according to the treatment protocols widely used in North America [33]. Our pediatric hematology department, however, bases treatment on the BFM and NOPHO European protocols [15,16], according to which age alone is not a parameter in defining patients as high risk of AL, and does not, therefore, affect the aggressiveness of anti-leukemia treatment protocols. A Japanese study, which analyzed 1991 admissions of 276 pediatric patients who received chemotherapy or HSCT, found age over 9 years was significantly associated with increased IFI rates. Patients in this study received antifungal prophylaxis based on baseline disease risk category [3]. The researchers hypothesized that this age-dependent susceptibility to IFI could be explained by a weaker immune defense associated with the atrophy of lymphoid tissue that occurs at approximately 10 years of age [34]. Another possible explanation is that older patients have more lifetime exposure and colonization with fungal spores, which may increase their IFI risk [35].

Although multivariable analysis did not associate AML and prolonged neutropenia with higher IFI risk, AML is a known risk factor of IFI [4], and we found a higher IFI rate in AML patients (37.0%; 10/27) than in those with ALL (18.2%, 12/66; *p* = 0.052). The Japanese study mentioned earlier suggests that the longer period of bone marrow suppression in AML treatment protocols, caused by more intensive chemotherapy protocols, is a possible cause of higher IFI rates in these patients [4]. It has also been shown that the risk for proven/probable IFD is increased in children who have undergone more intensive chemotherapy [32].

The main limitation of our study is its single center retrospective design. Our results cannot, therefore, be generalized to other centers and may be subject to bias. Specific local epidemiological features can, however, be better reflected in single-center studies. Comparison of FN episodes, rather than patients with and without IFI, enabled assessment of episode-specific characteristics. Our findings concerning the high rate of yeast IFI in traditional non-HR-IFI-AL groups challenge this classification and justify further investigation of the rate and risk factors for IFI in different AL pediatric patient populations in a prospective study.

## 5. Conclusions

In conclusion, the elevated risks of proven/probable mold IFI in HR-IFI-AL children associated with a substantial high mortality, and the high rate of invasive candidiasis in the non-HR-IFI-AL group emphasize the importance of reassessment of our center’s existing anti-fungal prophylaxis policy. Moreover, it is evident that the local rates and epidemiology of IFI should be monitored in each center to optimize patient management, based on current recommendations. In centers that do not use anti-mold prophylaxis, such as ours, close monitoring of local mold IFI rates, with the initiation of anti-mold prophylaxis when an increase in rate is expected, is important. Factors associated with increased risks of IFI, such as genetic syndromes, older age, presence of oral mucositis, and induction chemotherapy, can assist in targeting prophylaxis in groups at highest risk for IFI. This will hopefully improve management of these children and their prognosis.

## Figures and Tables

**Table 1:** Rates of proven or probable IFI in pediatric AL patients per chemotherapy stage and IFI risk category (n = 93).

IFI	IFI Risk Category *	Chemotherapy Stage	Proven or Probable IFI Rates ^1^ No. (%)
**All IFIs**	**HR-IFI-AL** (n = 52)	Induction chemotherapy	8 (15.4)
		All other chemotherapy stages	5 (9.6)
		Total	13 (25.0)
	**Non-HR-IFI-AL** (n = 41)	Induction chemotherapy	2 (4.9)
		All other chemotherapy stages	4 (9.8)
		Total	6 (14.6)
**Yeast**	**HR-IFI-AL** (n = 52)	Induction chemotherapy	4 (7.7)
		All other chemotherapy stages	2 (3.8)
		Total	6 (11.5)
	**Non-HR-IFI-AL** (n = 41)	Induction chemotherapy	2 (4.9)
		All other chemotherapy stages	3 (7.3)
		Total	5 (12.2)
**Mold** ^2^	**HR-IFI-AL** (n = 52)	Induction chemotherapy	4 (7.7)
		All other chemotherapy stages	3 (5.8)
		Total	7 (13.5)
	**Non-HR-IFI-AL** (n = 41)	Induction chemotherapy	0 (0.0)
		All other chemotherapy stages	1 (2.4)
		Total	1 (2.4)

Abbreviations: HR—high risk, AL—Acute Leukemia, IFI—invasive fungal infection, * HR-IFI-AL– includes AML, high-risk ALL (based on oncological risk categories) and relapsed patients, non-HR-IFI-AL– includes non-HR ALL (based on oncological risk categories). ^1^ Three patients that had two IFIs (yeast and mold), appear twice in this table. ^2^ *p* = 0.054 for comparison of the mold infections rate in HR-IFI-AL and non-HR-IFI-AL groups.

**Table 2:** Characteristics of FN episodes, and comparison between episodes with and without IFI (proven and probable).

Variable	All FN Episodes (n = 339)	FN Episodes without IFI Diagnosed (n = 321)	FN Episodes with Proven or Probable IFI Diagnosed (n = 18)	*p*-Value
**Sex** No. (%)				0.203
Female	196 (57.8)	183 (57.0)	13 (72.2)	
Male	143 (42.2)	138 (43.0)	5 (27.8)	
**Origin** No. (%)				0.134
Jewish	136 (40.1)	131 (40.8)	5 (27.8)	
Muslim	199 (58.7)	187 (58.3)	12 (66.7)	
Other	4 (1.2)	3 (0.9)	1 (5.6)	
**Chronic diseases** ^1^				0.114
No. (%)	23 (6.8)	20 (6.2)	3 (16.7)	
**Genetic syndromes** ^2^				**0.028**
No. (%)	35 (10.3)	30 (9.3)	5 (27.8)	
**AL type** No. (%)				**0.022**
ALL	254 (74.9)	245 (76.3)	9 (50)	
AML	85 (25.1)	76 (23.7)	9 (50)	
Age at AL diagnosis * (years)				0.053
Mean	5.7	5.6	7.6	
(SD)	(4.2)	(4.2)	(4.8)	
Age at start of FN episode * (years) Mean (SD)	6.7 (4.3)	6.6 (4.3)	7.8 (4.7)	0.243
Time since AL diagnosis ** (months) Median (IQR)	4.8 (2.2–9.5)	5.1 (2.3–9.7)	2.2 (0.7–4.0)	**0.002**
**Refractory disease**				0.281
No. (%)	6 (1.8)	5 (1.6)	1 (5.6)	
**Stage of treatment** No. (%)				**0.001**
Induction	63 (18.6)	53 (16.5)	10 (55.6)	
Consolidation Intensification Maintenance	227 (67)	220 (68.5)	7 (38.9)	
Relapse	49 (14.5)	48 (15.0)	1 (5.6)	
Length of FN episode * (days)				0.084
Mean	11.5	11.3	15.8	
(SD)	(10.9)	(10.9)	(9.2)	
**Febrile at the end of episode** ^3^ No. (%)	42 (12.4)	30 (9.3)	12 (70.6)	**<0.001**
**Breakthrough fever** No. (%)	92 (27.1)	82 (25.5)	10 (55.6)	**0.011**
**Oral mucositis** No. (%)	88 (26)	75 (23.4)	13 (76.5)	**<0.001**
**Diarrhea** No. (%)	89 (26.3)	80 (24.9)	9 (52.9)	**0.020**
**Presence of central line** No. (%)				0.154
No	8 (2.4)	8 (2.5)	0 (0.0)	
PICC	14 (4.1)	13 (4.0)	1 (5.6)	
Hickman	95 (28)	86 (26.8)	9 (50.0)	
Port	222 (65.5)	214 (66.7)	8 (44.4)	
**Steroids during 30 days before FN episode** No. (%) ^4^	192 (58.7)	177 (57.3)	15 (83.3)	**0.029**
**Antifungal treatment given from beginning of episode** No. (%)	147 (43.4)	140 (43.6)	7 (38.9)	0.694

Abbreviations: AL—acute leukemia, ALL—acute lymphocytic leukemia, AML—acute myeloid leukemia, FN—febrile neutropenia, central line, PICC—peripherally inserted central catheter. ^1^ Chronic disease—obesity, West syndrome, congenital heart disease, asthma, intellectual disability. ^2^ Genetic syndromes—Down’s syndrome (20 episodes), paraganglioma syndrome (one episode), Klinefelter syndrome (one episode), 1Q44 microdeletion syndrome (seven episodes), RUNX1 mutation (six episodes). ^3^ of 338 episodes (321 without IFI, 17 with IFI)—missing data. ^4^ of 327 episodes (309 without IFI, 18 with IFI)—missing data. * two-sample *t*-test was used. ** Mann–Whitney non parametric test.

**Table 3:** Univariable and multivariable analysis of factors associated with proven or probable IFI during a FN episode.

	Univariable Analysis			Multivariable Analysis		
	OR	95% CI	*p* Value	OR	95% CI	*p* Value
Genetic syndromes	3.287	1.210–8.934	0.020	11.513	2.555–51.875	0.001
Presence of oral mucositis	5.066	2.108–12.177	<0.001	15.364	4.134–57.105	<0.001
Time since AL diagnosis (months)	0.859	0.751–0.982	0.026	0.872	0.768–0.990	0.035
Age at AL diagnosis	1.076	0.985–1.176	0.105	1.138	1.004–1.289	0.043

Abbreviations: IFI—invasive fungal infections, AL—acute leukemia, FN—febrile neutropenia.

## Data Availability

The data presented in this study are available on reasonable request from the corresponding author.

## Supplementary Materials

The following supporting information can be downloaded at: https://www.mdpi.com/article/10.3390/microorganisms12010145/s1, Figure S1: (a) Total children with or without invasive fungal infections. (b) Total febrile neutropenic episodes with or without invasive fungal infections. (c) Total children with or without proven or probable invasive fungal infections. (d) Total febrile neutropenic episodes with or without proven or probable invasive fungal infections. Figure S2: Proven/probable invasive fungal infections over the study years. Table S1: Background data on children with acute leukemia. Table S2: Rate of at least one invasive fungal infection in children with acute leukemia. Table S3: Description of invasive fungal infections.

## References

[1] Groll A.H., Castagnola E., Cesaro S., Dalle J.H., Engelhard D., Hope W., Roilides E., Styczynski J., Warris A., Lehrnbecher T. (2014). Fourth European Conference on Infections in Leukaemia (ECIL-4): Guidelines for diagnosis, prevention, and treatment of invasive fungal diseases in paediatric patients with cancer or allogeneic haemopoietic stem-cell transplantation. Lancet Oncol..

[2] Cesaro S., Tridello G., Castagnola E., Calore E., Carraro F., Mariotti I., Colombini A., Perruccio K., Decembrino N., Russo G. (2017). Retrospective study on the incidence and outcome of proven and probable invasive fungal infections in high-risk pediatric onco-hematological patients. Eur. J. Haematol..

[3] Kobayashi R., Hori D., Sano H., Suzuki D., Kishimoto K., Kobayashi K. (2018). Risk Factors for Invasive Fungal Infection in Children and Adolescents with Hematologic and Malignant Diseases: A 10-year Analysis in a Single Institute in Japan. Pediatr. Infect. Dis. J..

[4] Kobayashi R., Kaneda M., Sato T., Ichikawa M., Suzuki D., Ariga T. (2008). The clinical feature of invasive fungal infection in pediatric patients with hematologic and malignant diseases: A 10-year analysis at a single institution at Japan. J. Pediatr. Hematol. Oncol..

[5] Lehrnbecher T., SchÃ¶ning S., Poyer F., Georg J., Becker A., Gordon K., Attarbaschi A., Groll A.H. (2019). Incidence and Outcome of Invasive Fungal Diseases in Children with Hematological Malignancies and/or Allogeneic Hematopoietic Stem Cell Transplantation: Results of a Prospective Multicenter Study. Front. Microbiol..

[6] Mor M., Gilad G., Kornreich L., Fisher S., Yaniv I., Levy I. (2011). Invasive fungal infections in pediatric oncology. Pediatr. Blood Cancer.

[7] Rosen G.P., Nielsen K., Glenn S., Abelson J., Deville J., Moore T.B. (2005). Invasive fungal infections in pediatric oncology patients: 11-year experience at a single institution. J. Pediatr. Hematol. Oncol..

[8] Sezgin Evim M., Tüfekçi Ö., Baytan B., Ören H., Çelebi S., Ener B., Üstün Elmas K., Yılmaz Ş., Erdem M., Hacımustafaoğlu M.K. (2022). Invasive Fungal Infections in Children with Leukemia: Clinical Features and Prognosis. Turk. J. Haematol..

[9] Lehrnbecher T., Robinson P., Fisher B., Alexander S., Ammann R.A., Beauchemin M., Carlesse F., Groll A.H., Haeusler G.M., Santolaya M. (2017). Guideline for the Management of Fever and Neutropenia in Children with Cancer and Hematopoietic Stem-Cell Transplantation Recipients: 2017 Update. J. Clin. Oncol..

[10] Riwes M.M., Wingard J.R. (2012). Diagnostic methods for invasive fungal diseases in patients with hematologic malignancies. Expert. Rev. Hematol..

[11] Hahn-Ast C., Glasmacher A., Mückter S., Schmitz A., Kraemer A., Marklein G., Brossart P., von Lilienfeld-Toal M. (2010). Overall survival and fungal infection-related mortality in patients with invasive fungal infection and neutropenia after myelosuppressive chemotherapy in a tertiary care centre from 1995 to 2006. J. Antimicrob. Chemother..

[12] Donnelly J.P., Chen S.C., Kauffman C.A., Steinbach W.J., Baddley J.W., Verweij P.E., Clancy C.J., Wingard J.R., Lockhart S.R., Groll A.H. (2020). Revision and Update of the Consensus Definitions of Invasive Fungal Disease from the European Organization for Research and Treatment of Cancer and the Mycoses Study Group Education and Research Consortium. Clin. Infect. Dis..

[13] Lehrnbecher T., Groll A.H. (2019). Pre-emptive versus empirical antifungal therapy in immunocompromised children. Lancet Child. Adolesc. Health.

[14] Freifeld A.G., Bow E.J., Sepkowitz K.A., Boeckh M.J., Ito J.I., Mullen C.A., Raad I.I., Rolston K.V., Young J.A., Wingard J.R. (2011). Clinical practice guideline for the use of antimicrobial agents in neutropenic patients with cancer: 2010 Update by the Infectious Diseases Society of America. Clin. Infect. Dis..

[15] Martin S. (2017). Treatment Protocol for Children and Adolescents with Acute Lymphoblastic Leukemia—AIEOP-BFM ALL 2017.

[16] Abrahamsson J. Research Study for Treatment of Children and Adolescents with Acute Myeloid Leukaemia 0–18 Years (AML2012). https://www.clinicaltrials.gov/study/NCT01828489.

[17] Groll A.H., Pana D., Lanternier F., Mesini A., Ammann R.A., Averbuch D., Castagnola E., Cesaro S., Engelhard D., Garcia-Vidal C. (2021). 8th European Conference on Infections in Leukaemia: 2020 guidelines for the diagnosis, prevention, and treatment of invasive fungal diseases in paediatric patients with cancer or post-haematopoietic cell transplantation. Lancet Oncol..

[18] De Pauw B., Walsh T.J., Donnelly J.P., Stevens D.A., Edwards J.E., Calandra T., Pappas P.G., Maertens J., Lortholary O., Kauffman C.A. (2008). Revised definitions of invasive fungal disease from the European Organization for Research and Treatment of Cancer/Invasive Fungal Infections Cooperative Group and the National Institute of Allergy and Infectious Diseases Mycoses Study Group (EORTC/MSG) Consensus Group. Clin. Infect. Dis..

[19] Lehrnbecher T., Groll A.H., Cesaro S., Alten J., Attarbaschi A., Barbaric D., Bodmer N., Conter V., Izraeli S., Mann G. (2023). Invasive fungal diseases impact on outcome of childhood ALL—An analysis of the international trial AIEOP-BFM ALL 2009. Leukemia.

[20] Cornely O.A., Maertens J., Winston D.J., Perfect J., Ullmann A.J., Walsh T.J., Helfgott D., Holowiecki J., Stockelberg D., Goh Y.T. (2007). Posaconazole vs. fluconazole or itraconazole prophylaxis in patients with neutropenia. N. Engl. J. Med..

[21] Fisher B.T., Zaoutis T., Dvorak C.C., Nieder M., Zerr D., Wingard J.R., Callahan C., Villaluna D., Chen L., Dang H. (2019). Effect of Caspofungin vs Fluconazole Prophylaxis on Invasive Fungal Disease Among Children and Young Adults with Acute Myeloid Leukemia: A Randomized Clinical Trial. JAMA.

[22] Santolaya M.E., Alvarez A.M., Acuna M., Aviles C.L., Salgado C., Tordecilla J., Varas M., Venegas M., Villarroel M., Zubieta M. (2018). Efficacy of pre-emptive versus empirical antifungal therapy in children with cancer and high-risk febrile neutropenia: A randomized clinical trial. J. Antimicrob. Chemother..

[23] Maertens J., Lodewyck T., Donnelly J.P., Chantepie S., Robin C., Blijlevens N., Turlure P., Selleslag D., Baron F., Aoun M. (2023). Empiric vs Preemptive Antifungal Strategy in High-Risk Neutropenic Patients on Fluconazole Prophylaxis: A Randomized Trial of the European Organization for Research and Treatment of Cancer. Clin. Infect. Dis..

[24] Yeoh D.K., Moore A.S., Kotecha R.S., Bartlett A.W., Ryan A.L., Cann M.P., McMullan B.J., Thursky K., Slavin M., Blyth C.C. (2021). Invasive fungal disease in children with acute myeloid leukaemia: An Australian multicentre 10-year review. Pediatr. Blood Cancer.

[25] Douglas A.P., Stewart A.G., Halliday C.L., Chen S.C. (2023). Outbreaks of Fungal Infections in Hospitals: Epidemiology, Detection, and Management. J. Fungi.

[26] Zawitkowska J., Drabko K., Szmydki-Baran A., Zaucha-Prazmo A., Lejman M., Czyzewski K., Zalas-Wiecek P., Gryniewicz-Kwiatkowska O., Czajnska-Deptula A., Kulicka E. (2019). Infectious profile in children with ALL during chemotherapy: A report of study group for infections. J. Infect. Chemother. Off. J. Jpn. Soc. Chemother..

[27] Sahbudak Bal Z., Yilmaz Karapinar D., Karadas N., Sen S., Onder Sivis Z., Akinci A.B., Balkan C., Kavakli K., Vardar F., Aydinok Y. (2015). Proven and probable invasive fungal infections in children with acute lymphoblastic leukaemia: Results from an university hospital, 2005–2013. Mycoses.

[28] Whitlock J.A., Sather H.N., Gaynon P., Robison L.L., Wells R.J., Trigg M., Heerema N.A., Bhatia S. (2005). Clinical characteristics and outcome of children with Down syndrome and acute lymphoblastic leukemia: A Children’s Cancer Group study. Blood.

[29] Dordelmann M., Schrappe M., Reiter A., Zimmermann M., Graf N., Schott G., Lampert F., Harbott J., Niemeyer C., Ritter J. (1998). Down’s syndrome in childhood acute lymphoblastic leukemia: Clinical characteristics and treatment outcome in four consecutive BFM trials. Berlin-Frankfurt-Munster Group. Leukemia.

[30] Eissa S., Khedr R., Romeih M., Halaby L., Elanany M., Madney Y. (2022). Clinical characteristics and outcome of invasive fungal sinusitis in children with hematological malignancies. Med. Mycol..

[31] Hovi L., Saxen H., Saarinen-Pihkala U.M., Vettenranta K., Meri T., Richardson M. (2007). Prevention and monitoring of invasive fungal infections in pediatric patients with cancer and hematologic disorders. Pediatr. Blood Cancer.

[32] O’Reilly M.A., Govender D., Kirkwood A.A., Vora A., Samarasinghe S., Khwaja A., Grandage V., Rao A., Ancliff P., Pavasovic V. (2019). The incidence of invasive fungal infections in children, adolescents and young adults with acute lymphoblastic leukaemia/lymphoma treated with the UKALL2011 protocol: A multicentre retrospective study. Br. J. Haematol..

[33] Bertolone K.L., Landier W., Baggott C., Fochtman D., Foley G., Patterson Kelly K. (2011). Acute lymphoblastic leukemia. Nursing Care of Children and Adolsecents with Cancer and Blood Disorders.

[34] Scammon R.E. (1930). The measurement of the body in childhood. The Measurement of Man, Harris, J.A., Jackson, C.M., Paterson, D.G., Scammon, R.E., Eds..

[35] Dini G., Castagnola E., Comoli P., van Tol M.J., Vossen J.M. (2001). Infections after stem cell transplantation in children: State of the art and recommendations. Bone Marrow Transplant..

