# Supplementary material for: Invasive Fungal Infections in Children with Acute Leukemia: Epidemiology, Risk Factors, and Outcome
Source: Microorganisms. 2024 Jan 11;12(1):145. doi: 10.3390/microorganisms12010145 (PMC10820110; doi:10.3390/microorganisms12010145)

Supplemental Materials

Table S1. Background data on children with acute leukemia

**Variable**	**Number (%) (n=93)**
**Sex**	
Female	51 (54.8)
Male	42 (45.2)
**Age at AL diagnosis in years Median (IQR)**	4.4 (2.8-9.2)
**Origin**	
Jewish	36 (38.7)
Muslim	55 (59.1)
Other	2 (2.2)
**Chronic diseases ***	5 (5.4)
**Genetic syndromes ****	9 (9.7)
**AL type**	
ALL	66 (71)
AML	27 (29)
**Relapsed Disease**	9 (9.7)
**Refractory Disease**	3 (3.2)

Abbreviations: AL – acute leukemia, ALL – acute lymphocytic leukemia, AML – acute myeloid leukemia, FN – febrile neutropenia.

* Chronic diseases – obesity, West syndrome, congenital heart disease, asthma, intellectual disability.

** Genetic syndromes – Down Syndrome (5 patients), paraganglioma syndrome (1 patient), Klinefelter syndrome (1 patient), 1Q44 microdeletion syndrome (1 patient), RUNX1 mutation (1 patient).

Table S2. Rate of at least one invasive fungal infection in children with acute leukemia

**Infection type**	**Number (percentage)***	**Rate ALL (percentage)**	**Rate AML (percentage)**
Number of children	93	66	27
Total IFI	22 (23.7)	12 (18.2%)	10 (37.0%)
Proven or probable IFI	17 (18.3)	9 (13.6%)	8 (29.7%)
Possible IFI	6 (6.5%)	3 (4.6%)	3 (11.1%)
Yeast IFI	12 (12.9)	7 (10.6%)	5 (18.5%)
Proven or probable yeast IFI	11 (11.8)	7 (10.6%)	4 (14.8%)
Possible yeast IFI	1 (1.1%)	0	1 (3.7%)
Mold IFI	13 (14.0)	6 (9.1%)	7 (25.9%)
Proven or probable mold IFI	8 (8.6)	3 (4.6%)	5 (18.5%)
Possible mold IFI	5 (5.4%)	3 (4.6%)	2 (7.4%)

Abbreviations: ALL – acute lymphocytic leukemia, AML – acute myeloid leukemia, IFI – invasive fungal infections.

*3 patients that had 2 IFIs (yeast and mold), appear twice in this table in relevant categories

Table S3. Description of invasive fungal infections

IFI episode number	Gender	Age (years)	Underlying disease (stage of treatment)	IFI risk*	IFI definition	Description and susceptibility	Break-through (antifungal agent)	Treatment +duration	Outcome IFI	Outcome at last follow up (months since IFI until last follow up)
1	Female	4.6	ALL SR (I)	non-HR-IFI	Proven yeast	Candidemia (*C. albicans*), lesions in kidneys, rt eye, soft tissue, spleen	no	F (4 months) + V (one intraophthalmic) + prednisone (3 weeks), continued secondary F prophylaxis	CR	Alive (16)
2	Female	2.8	AML SR (Co)	HR-IFI	Proven yeast	Candidemia (*C. glabrata*)	yes (F)	C (4 days) -> F (10 days).	CR	Alive (20)
3	Female	3.5	ALL SR (Co)	non-HR-IFI	Proven yeast	Hepatosplenic candidiasis on imaging, liver biopsy revealed yeasts, culture negative	yes (V)	F (3.5 months until lesions resolved)	CR	Alive (36)
4	Male	6.7	AML SR (I)	HR-IFI	Probable mold	CT lung findings; GM>1 in blood and BAL	yes (F)	Mostly V, few days Am B (11 months total until immunosuppression stopped and CT lesions resolved)	CR	Alive (28)
5	Female	13	AML HR (Co)	HR-IFI	Proven mold	Gum necrotic lesion, bone biopsy with hyphae compatible with aspergillosis, culture negative	yes (F)	Is (mostly), V/It/Am B (few days), (total 3 months until the end of immune suppression)	CR	Alive (43)
6	Female	1.6	AML SR (I)	HR-IFI	Possible mold	CT lung findings, BAL not performed, blood GM negative	yes (F)	V (4.5 months until the end of chemotherapy)	CR	Alive (6)
7	Male	4.4	AML SR (I)	HR-IFI	Probable mold	CT lung findings; GM>1 in blood and BAL	yes (F)	V (5 months until the end of chemotherapy)	CR	Alive (40)

8	Male	10.7	AML SR (I)	HR-IFI	Proven yeast	Candidemia (*C. tropicalis*), lesions in spleen, liver, kidney, lymph nodes	no	F (total 27 months), C + Am B (2 months), An (1.5 months) + splenectomy + steroids (3 months)	CR	Alive (44)
9	Male	14.9	AML SR (Co)	HR-IFI	Possible yeast	Lesions compatible with hepatosplenic candidiasis on imaging, biopsy not performed	no	C (few days), F (3 months + secondary prophylaxis until death).	CR	Death (7) due to refractory AL
10	Female	15	ALL SR (Co)	non-HR-IFI	Proven yeast	Kidney and liver lesions on imaging, kidney biopsy revealed *Candida albicans*	no	F (6 months), nephrostomy	CR	Alive (54)
11[1]	Female	1.4	ALL HR (RI)	HR-IFI	Probable mold	CT lung findings, blood GM>1; GM negative in BAL	yes (F)	V (17 days) then Am B (8 days until death)	Death	Death (0.83) IFI probably contributed
12[1]	Female	1.4	ALL HR (RI)	HR-IFI	Proven yeast	Candidemia (*C. glabrata*), lesions in spleen, liver, kidney	yes (V)	Am B (8 days until death)	Death	Death (0.25) IFI probably contributed
13	Female	4.9	ALL MR (I)	non-HR-IFI	Proven yeast	Candidemia (*C. albicans*)	no	An (3 weeks)	CR	Alive (68)
14	Female	3.5	ALL HR (Re)	HR-IFI	Proven mold	Lung and sinuses involvement, *Fusarium solani* complex cultured from blood+positive GM (>1) in BAL and blood, skin lesions with hyphae on biopsy	yes (F)	V (2 months) + concomitant Am B (5 days) until death	Death	Death (2) relapse ALL
15	Female	15	AML SR (I)	HR-IFI	Proven mold	Disseminated lesions in brain, liver, spleen, lungs; kidney biopsy with hyphae compatible with mucormycosis	yes (F)	An (1 week), Lam (few days until death)	Death	Death (0.5) IFI probably contributed
16	Female	7.4	ALL SR (In)	non-HR-IFI	Probable mold	CT lung findings+*Aspergillus niger* cultured from BAL+GM > 1 in BAL, GM negative in blood	no	V (2.5 months)	CR	Alive (44)

17	Male	12.5	ALL SR (In)	non-HR-IFI	Proven yeast	multiple abscesses in muscles and liver, *Candida tropicalis* cultured from muscle biopsy	no	F (6 months)	CR	Alive (46)
18	Male	2.8	ALL HR (I)	HR-IFI	Proven yeast	Necrotizing soft tissue infection, *Candida glabrata* cultured from biopsy	no	F (2.5 months)	CR	Alive (48)
19[2]	Female	9.4	AML HR (I)	HR-IFI	Proven yeast	Candidemia (*Candida dubliniensis*), hepatosplenic candidiasis with yeasts seen in liver biopsy	yes (mainly F)	C (few days)-->F (7 weeks)-->V (suspected lung mold IFI) + steroids in 2 courses (80 days and 85 days)	Partial response (recovered from candidemia but recovery from hepatosplenic candidiasis can't be determined because she developed another IFI and another biopsy was not done)	Death (22) due to relapse AML
20[2]	Female	9.4	AML HR (I)	HR-IFI	Proven mold	CT findings, lung biopsy compatible with mucormycosis, culture negative	yes (F)	P (mostly); V or Am B (few days)	CR	Death (22) due to relapse AML
21[3]	Female	0.3	AML SR (I)	HR-IFI	Possible mold	CT lung findings, blood GM 0.69. BAL not performed	yes (F)	V (2 months)	CR	Death (12) due to refractory AML
22[3]	Female	0.3	AML SR (I)	HR-IFI	Proven yeast	Liver lesions, biopsy revealed yeasts, culture negative	yes (F)	F (10 months until death)	Progression (new lesion in liver after normal US), later died due to refractory AML	Death (10) due to refractory AML
23	Male	3.1	ALL HR (In)	HR-IFI	Possible mold	CT lung findings, negative blood GM, BAL not performed	no	V (5 weeks)	CR	Alive (5)
24	Female	4.4	ALL SR (Co)	non-HR-IFI	Possible mold[4]	CT lung findings, GM in BAL (0.8) and blood (0.5)	no	V (3 months)	CR	Alive (8)
25	Male	9	ALL HR (Co)	HR-IFI	Possible mold[4]	CT lung findings, GM in blood (value 0.8); negative GM in BAL	yes (F)	V (3 months)	CR	Alive (19)

Abbreviations: ALL – acute lymphocytic leukemia, AML – acute myeloid leukemia, BAL – bronchoalveolar lavage, CR – complete recovery, CT- computerized tomography, GM – galactomannan, HR-high risk, IFI – invasive fungal infection, SR – standard risk

Antifungal agents: Am B– amphotericin B, An – anidulafungin, C – caspofungin, F – fluconazole, Is – isavuconazole, It – itraconazole, Lam – liposomal amphotericin, M – micafungin, P – posaconazole, V- voriconazole

Stages of acute leukemia treatment: Co – consolidation, I – induction, In – intensification, Re – relapse, RI – reinduction.

[1] the same patient
[2] the same patient
[3] the same patient
[4] considered a possible and not probable mold infection as the value of GM did not reach the accepted cutoffs in blood/BAL

*risk for IFI according to ECIL criteria [17] – HR-IFI includes HR-ALL, AML and relapsed disease, non-HR-IFI includes standard and intermediate risk ALL

Figure S1a. Total children with or without invasive fungal infections

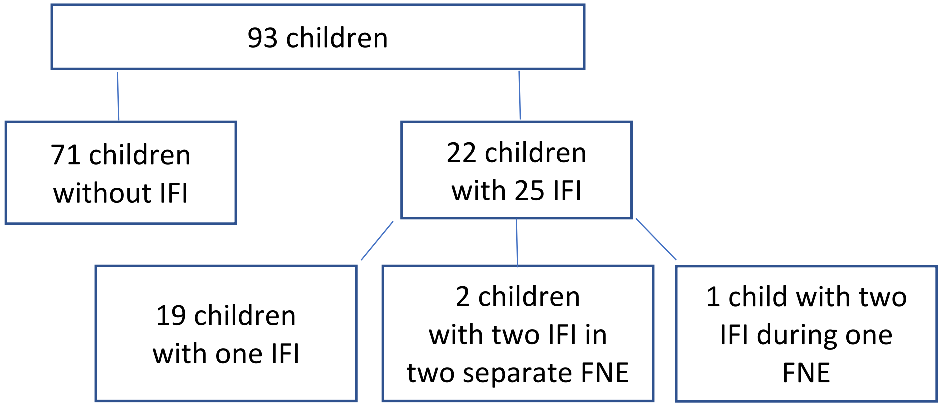

Figure S1b. Total febrile neutropenic episodes with or without invasive fungal infections

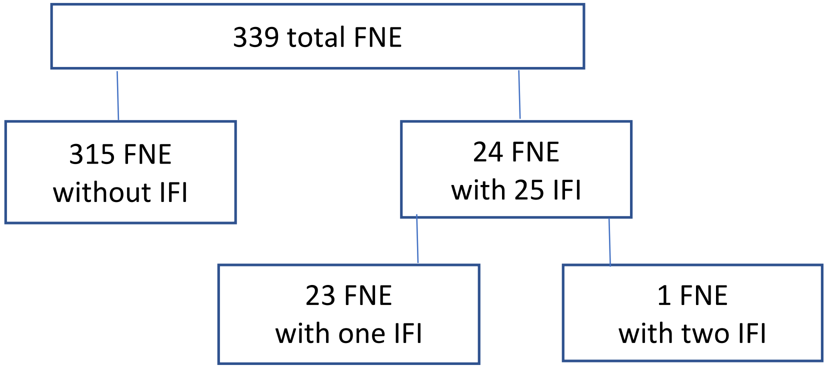

Figure S1c. Total children with or without proven or probable invasive fungal infections

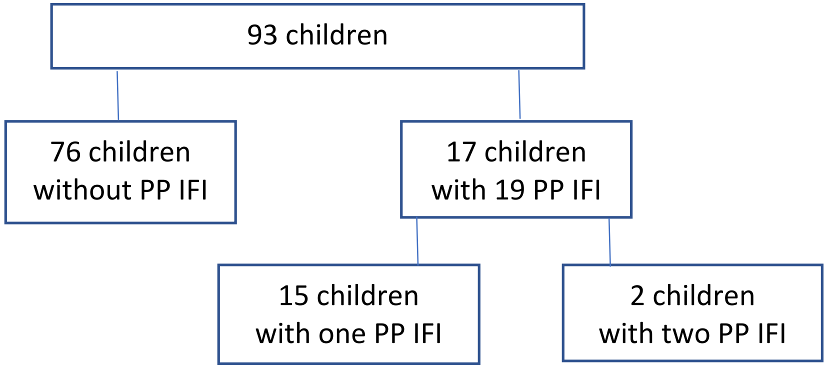

Figure S1d. Total febrile neutropenic episodes with or without proven or probable invasive fungal infections

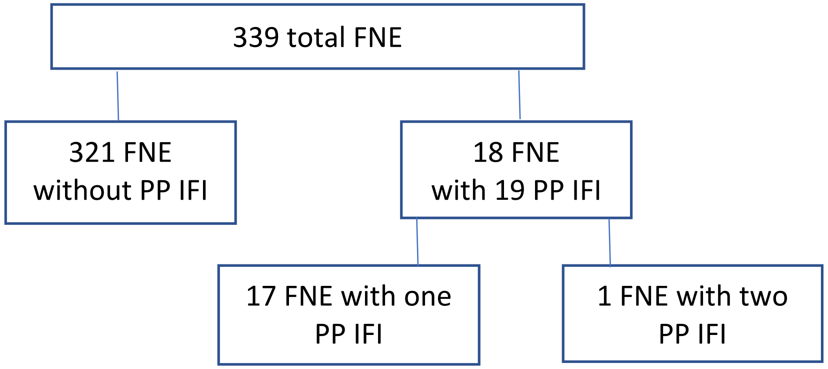

FNE – febrile neutropenic episodes
IFI - invasive fungal infections
PP – proven or probable IFI

Figure S2. Proven/probable invasive fungal infections over the study years

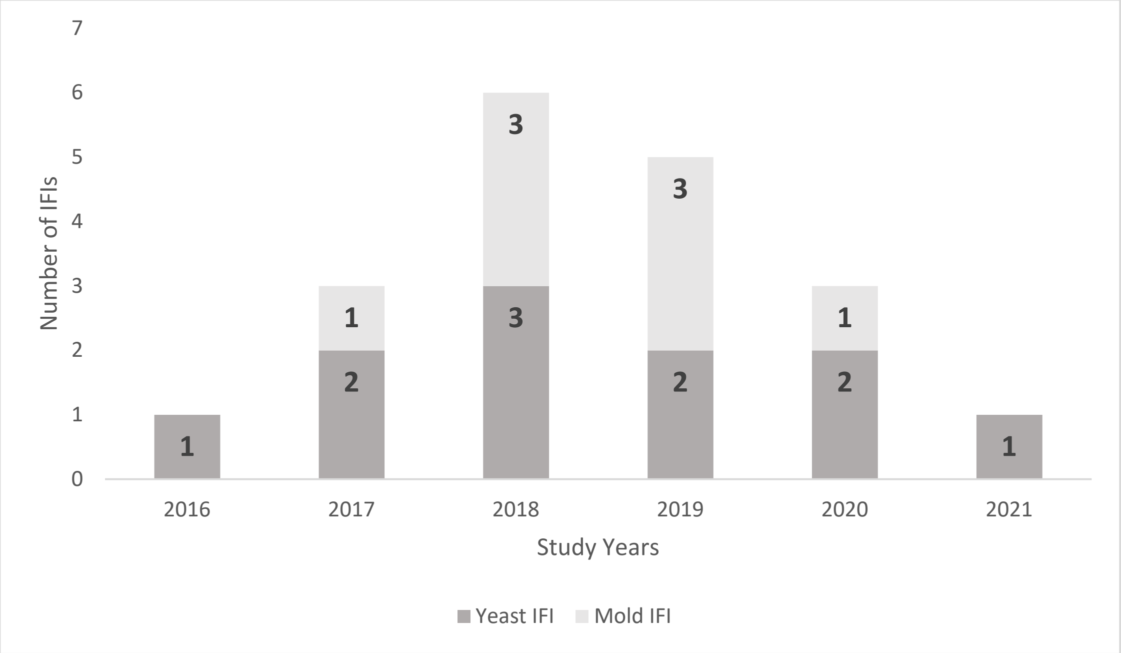

Supplement: Supplementary file 1 [file microorganisms-12-00145-s001.zip › microorganisms-2806285-supplementary.pdf]
